# Supplementary material for: Dorsal-Ventral Differences in Neural Stem Cell Quiescence Are Induced by p57KIP2/Dacapo
Source: Dev Cell. 2019 Apr 22;49(2):293–300.e3. doi: 10.1016/j.devcel.2019.02.015 (PMC6486397; doi:10.1016/j.devcel.2019.02.015)
Supplement: Document S1. Figures S1–S4 [file mmc1.pdf]

Developmental Cell, Volume *49*

Supplemental Information

Dorsal-Ventral Differences in Neural Stem Cell Quiescence Are Induced by $p57^{KIP2}$/Dacapo

Leo Otsuki and Andrea H. Brand

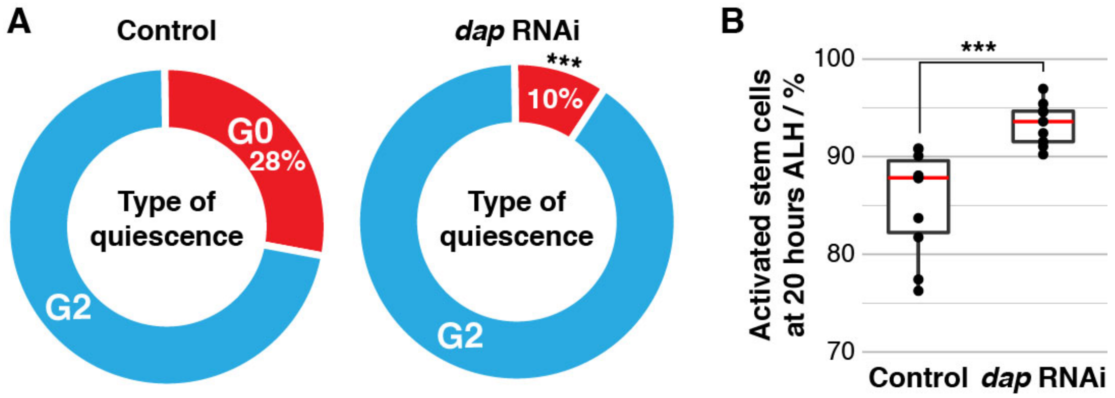

<u>Figure S1, Related to Figure 1. *dap* knockdown in NSCs leads to a switch from G_0 to G_2 quiescence and acceleration in stem cell activation timing</u>

(**A**) Proportions of G_0 and G_2 quiescent NSCs in control (*wor*-GAL4>*mCherry* RNAi) *versus* *dap* knockdown (*wor*-GAL4>*dap* RNAi) brains at 0ALH. Control: 28±1.0% G_0. *dap* knockdown: 10±0.5% G_0. *n*=10 tVNCs (control) or 7 tVNCs (knockdown), ~140 NSCs each. ***: $p=5.04\times10^{-10}$, Welch's test.

(**B**) Activated NSCs in control (*wor*-GAL4>*mCherry* RNAi) *versus dap* knockdown (*wor*-GAL4>*dap* RNAi) tVNCs at 20 hours ALH. Wor expression was used to label activated stem cells (Otsuki and Brand, 2018). *n*=10 tVNCs (control) or 9 tVNCs (RNAi), ~130 NSCs each. ***: $p=7.65\times10^{-4}$, Student's *t*-test.

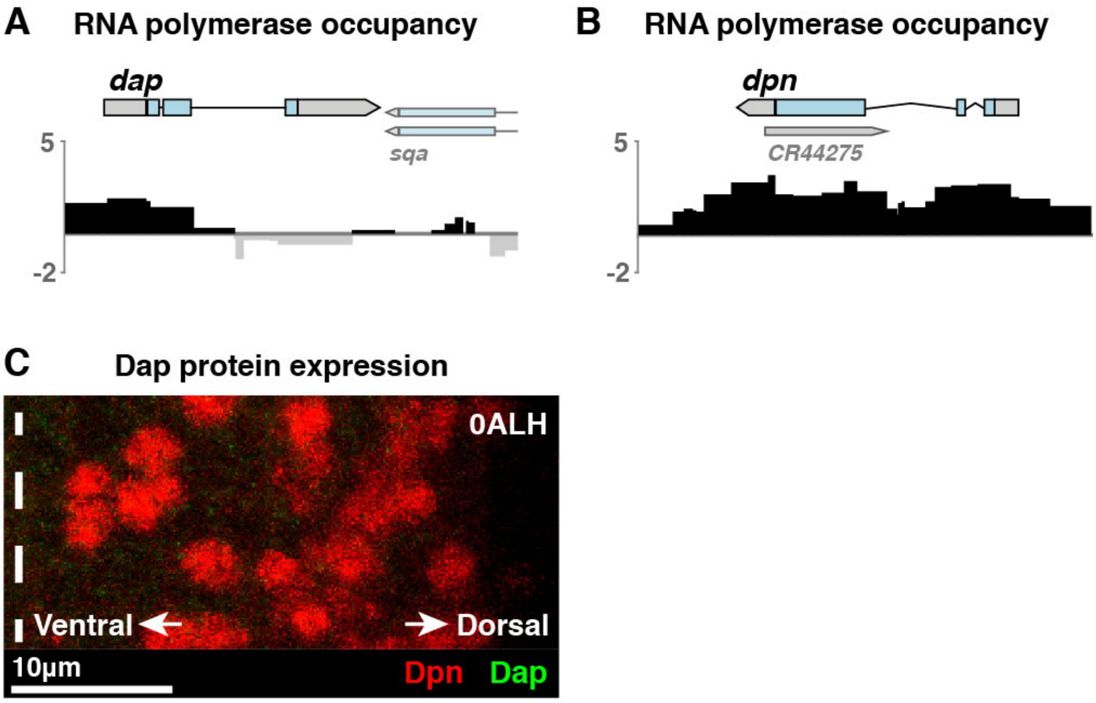

Figure S2, Related to Figure 2. Quiescent NSCs do not transcribe or translate *dap*

(**A-B**) RNA polymerase II occupancy at the *dap* and *dpn* loci in quiescent NSCs, as determined by Targeted DamID. RNA polymerase II is poised at the 5' end of the *dap* locus, but *dap* is not transcribed (A), in contrast to *dpn* which is actively transcribed in quiescent NSCs (B). Axis depicts log_2 ratio change between test and reference samples. Data from Otsuki and Brand, 2018.

(**C**) Quiescent NSCs do not express Dap protein at 0ALH. Single hemi-segment of a tVNC co-stained to visualise NSCs (red) and Dap (green) at 0ALH. Dotted line indicates the ventral midline. Anterior is up and dorsal is to the right. Maximum intensity projection.

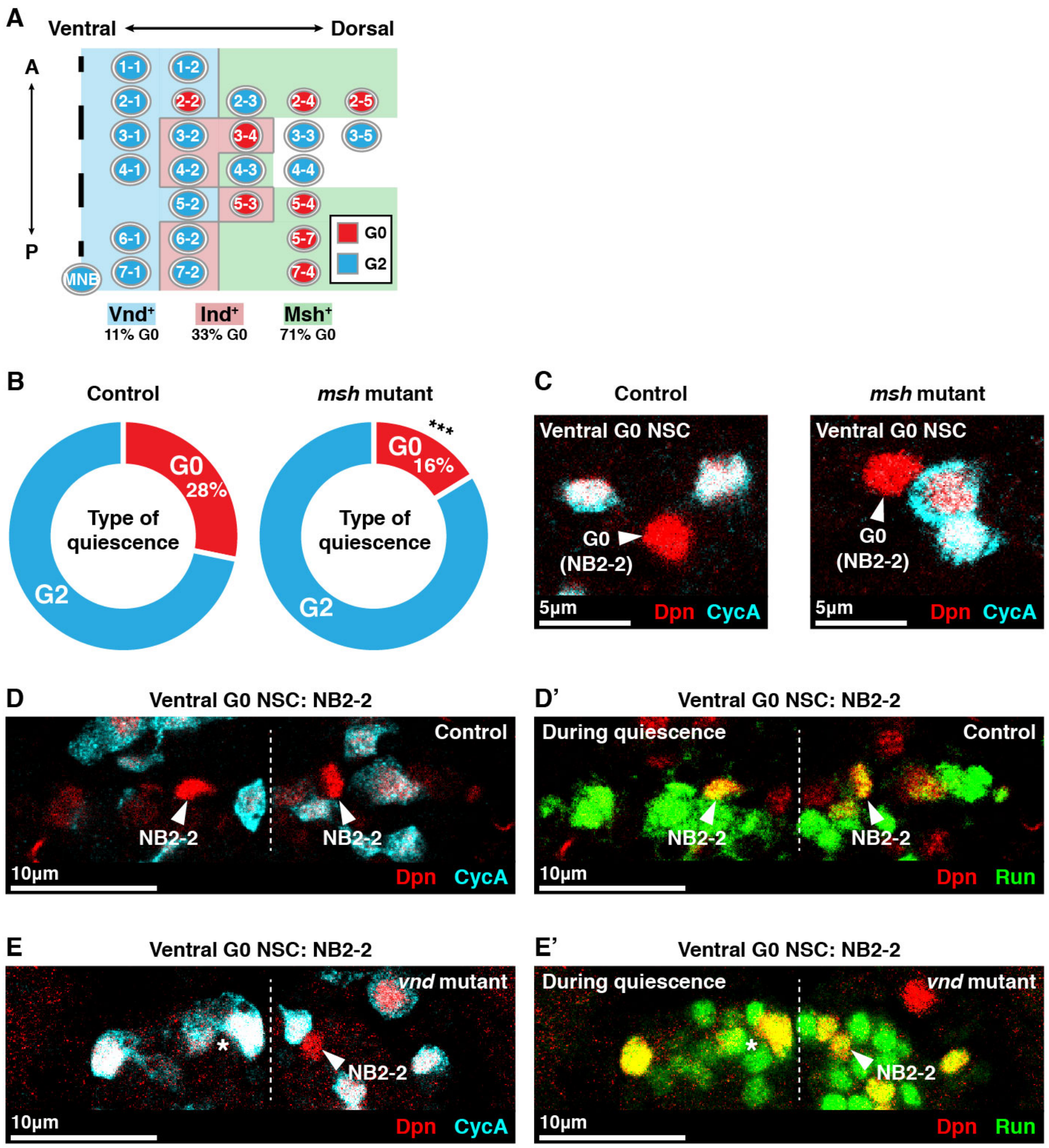

Figure S3, Related to Figure 3. Dorsal-ventral patterning of NSCs in the tVNC

(**A**) Map overlaying the distribution of G_0 (red) and G_2 (cyan) quiescent NSCs with the Msh^+ (pale green), Ind^+ (pale red) and Vnd^+ (pale blue) regions of the tVNC. Map assembled using data from (Chu et al., 1998; D'Alessio and Frasch, 1996; Isshiki et al., 1997; McDonald et al., 1998; Otsuki and Brand, 2018; Weiss et al., 1998). Dotted line indicates ventral midline. A: anterior. P: posterior.

(**B**) Proportions of G_0 and G_2 quiescent NSCs in control (*msh*$^{\Delta 68}$ heterozygous) *versus msh*$^{\Delta 68}$ mutant brains at stage 17 embryogenesis. *n*=10 tVNCs, ~140 NSCs each. ***: *p*=3.70x10^{-6}, Welch's test. Error bars indicate S.E.M.

(**C**) The ventral G_0 NSC NB2-2 (arrowed) still arrests in G_0 ($CycA^-$; red) in *msh* mutants. Single section confocal image.

(**D-D'**) In control embryos, the ventral G_0 NSC NB2-2 (arrowed) can be identified by its proximity to the midline (dotted line) and expression of the transcription factor Runt (Run, green). NB2-2 does not express CycA (cyan), identifying it as a G_0 NSC. Stage 17 embryo, single section confocal image.

(**E-E'**) NB2-2 is not always formed in *vnd*^{6} mutant embryos (Chu et al., 1998). However, in 33% of hemi-segments, a G_0 ($CycA^-$), Run^+ NSC is present close to the midline (dotted line), which we interpret to be the ventral G_0 NSC NB2-2. *n*=10 embryos, 6 hemi-segments each. Asterisk indicates expected position of NB2-2 in a hemi-segment lacking NB2-2. Stage 17 embryo, single section confocal image.

Anterior is up in all images.

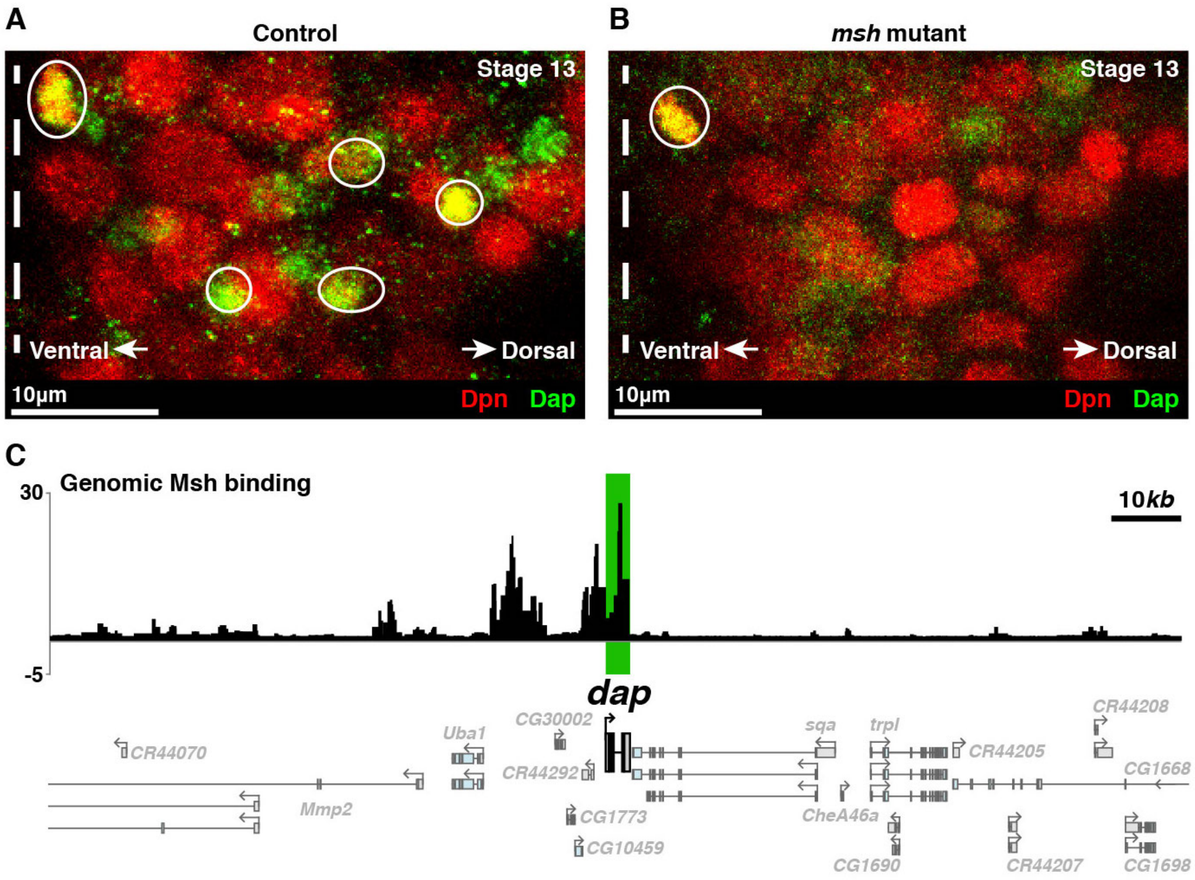

Figure S4, Related to Figure 4. Msh regulates Dap expression in NSCs

(**A**-**B**) Single hemi-segments from control (A) and *msh* mutant (B) tVNCs, co-stained to visualise NSCs (red) and Dap (green). Dap^{+} NSCs (yellow) are circled. Anterior is up; dorsal is right. Dorsal NSCs lose Dap expression in *msh* mutants. Dotted line indicates midline. Maximum intensity projections.

(**C**) Msh binding intensity across ~175kb of chromosome 2R, encompassing the *dap* locus (green box). Msh binds to the *dap* locus. Msh binding was assessed specifically in embryonic NSCs *in vivo* using TaDa (Southall et al., 2013). Unlogged data assembled from three biological replicates.
