# Supplementary material for: Dorsal-Ventral Differences in Neural Stem Cell Quiescence Are Induced by p57KIP2/Dacapo
Source: Dev Cell. 2019 Apr 22;49(2):293–300.e3. doi: 10.1016/j.devcel.2019.02.015 (PMC6486397; doi:10.1016/j.devcel.2019.02.015)
Supplement: Document S2. Article plus Supplemental Information [file mmc2.pdf]

Short Article

Developmental Cell

Dorsal-Ventral Differences in Neural Stem Cell Quiescence Are Induced by p57^{KIP2}/Dacapo

Graphical Abstract

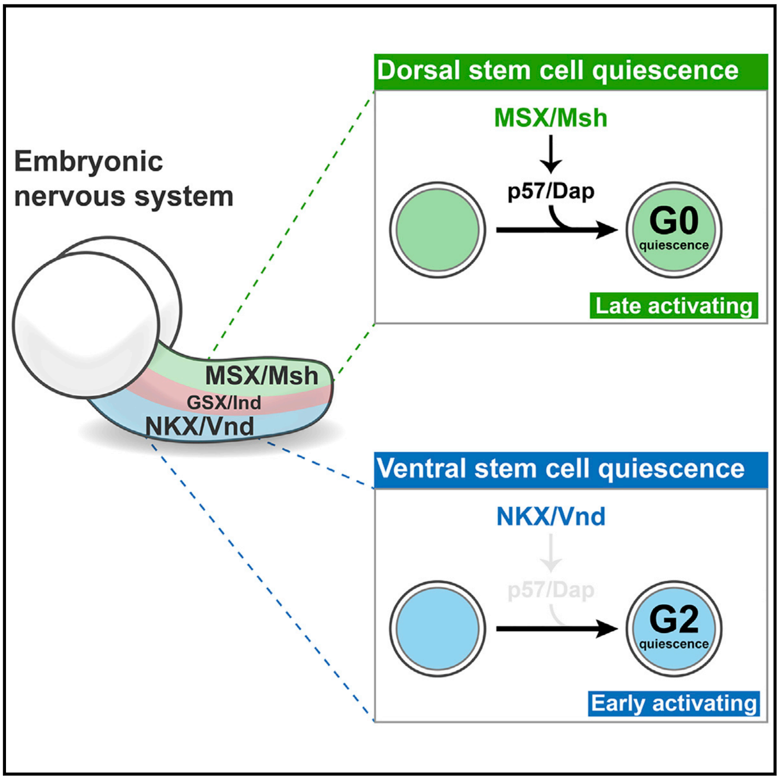

Authors

Leo Otsuki, Andrea H. Brand

Correspondence

a.brand@gurdon.cam.ac.uk

In Brief

Otsuki and Brand reveal that axis patterning during embryogenesis primes neural stem cells (NSCs) to undergo different types of quiescence. Dorsal NSCs express the patterning factor MSX/Msh, which triggers p57^{KIP2}/Dap expression and G_0 quiescence. Ventral NSCs, negative for MSX/Msh, undergo G_2 quiescence. G_2 cells become activated before G_0 cells post-embryonically.

Highlights

- p57/Dap determines whether neural stem cells enter G_0 quiescence or G_2 quiescence
- The dorsal patterning factor MSX/Msh promotes p57/Dap expression and G_0 quiescence
- Ventral stem cells instead express NKX/Vnd and undergo G_2 quiescence
- Stem cells undergo distinct types of quiescence depending on axial identity

Otsuki & Brand, 2019, Developmental Cell *49*, 293–300
April 22, 2019 © 2019 The Authors. Published by Elsevier Inc.
https://doi.org/10.1016/j.devcel.2019.02.015

Developmental Cell
Short Article

Dorsal-Ventral Differences in Neural Stem Cell Quiescence Are Induced by p57^{KIP2}/Dacapo

Leo Otsuki[1,2] and Andrea H. Brand[1,3,*]
[1]The Gurdon Institute and Department of Physiology, Development and Neuroscience, University of Cambridge, Tennis Court Road, Cambridge CB2 1QN, UK
[2]Present address: Research Institute of Molecular Pathology (IMP), Vienna Biocenter (VBC), Campus-Vienna-Biocenter 1, 1030 Vienna, Austria
[3]Lead Contact
*Correspondence: a.brand@gurdon.cam.ac.uk
https://doi.org/10.1016/j.devcel.2019.02.015

SUMMARY

Quiescent neural stem cells (NSCs) in the adult brain are regenerative cells that could be activated therapeutically to repair damage. It is becoming apparent that quiescent NSCs exhibit heterogeneity in their propensity for activation and in the progeny that they generate. We discovered recently that NSCs undergo quiescence in either G_0 or G_2 in the *Drosophila* brain, challenging the notion that all quiescent stem cells are G_0 arrested. We found that G_2-quiescent NSCs become activated prior to G_0 NSCs. Here, we show that the cyclin-dependent kinase inhibitor Dacapo (Dap; ortholog of p57^{KIP2}) determines whether NSCs enter G_0 or G_2 quiescence during embryogenesis. We demonstrate that the dorsal patterning factor, Muscle segment homeobox (Msh; ortholog of MSX1/2/3) binds directly to the Dap locus and induces Dap expression in dorsal NSCs, resulting in G_0 arrest, while more ventral NSCs undergo G_2 quiescence. Our results reveal region-specific regulation of stem cell quiescence.

INTRODUCTION

Neural stem cells (NSCs) are located in two main regions of the adult mammalian brain, the dentate gyrus of the hippocampus and the ventricular-subventricular zone (V-SVZ) of the lateral ventricles (Doetsch et al., 1999; Seri et al., 2001). These NSCs reside primarily in a mitotically dormant state known as quiescence. Stimuli including injury and exercise can induce quiescent NSCs to divide and produce new neurons or glia (Llorens-Bobadilla et al., 2015; Lugert et al., 2010). By characterizing quiescence regulators, it may become possible to activate quiescent NSCs on demand and regenerate brain tissue following injury or disease (Encinas and Fitzsimons, 2017).

Quiescent NSCs vary in their sensitivities or responses to external stimuli, suggesting that they undergo different types of quiescence. For example, "resting" stem cells in the adult mouse hippocampus, which are quiescent NSCs that have proliferated recently, are more likely to become activated than naive quiescent NSCs (Urbán et al., 2016). Quiescent progenitors are also differentially responsive to norepinephrine and KCl (Jhaveri et al., 2015). Once activated, quiescent NSCs in the brain generate different types of progeny in a region-specific manner (Fuentealba et al., 2015). Single-cell profiling has revealed transcriptional and metabolic heterogeneity in quiescent NSCs, linked to priming for activation and regional identity (Dulken et al., 2017; Llorens-Bobadilla et al., 2015; Shin et al., 2015). Although it is now appreciated that quiescent NSCs exhibit significant heterogeneity (Chaker et al., 2016), the factors that induce stem cells to undergo different types of quiescence in the brain are not understood well.

It has been widely accepted for many years that quiescent stem cells arrest in G_0 of the cell cycle (Cheung and Rando, 2013). However, we discovered recently that NSCs in the *Drosophila melanogaster* central nervous system undergo two distinct types of quiescence: 75% of NSCs arrest in G_2 of the cell cycle and only 25% arrest in G_0 (Otsuki and Brand, 2018). G_2-quiescent NSCs activate rapidly in response to a nutritional stimulus, while G_0-quiescent NSCs respond more slowly (Figure 1A) (Otsuki and Brand, 2018). Thus, G_2 and G_0 NSCs are functionally distinct types of quiescent stem cell. We showed that NSCs are pre-programmed to undergo G_0 or G_2 quiescence in an invariant manner (Otsuki and Brand, 2018). An understanding of the differential regulation of G_0/G_2 quiescence should reveal the factors that trigger different types of stem cell quiescence.

Here, we demonstrate that the cyclin-dependent kinase inhibitor p57/Dap directs NSCs to enter G_0 quiescence, rather than G_2 quiescence, during embryogenesis. Upon loss of *p57/dap*, NSCs switch from G_0 to G_2 quiescence and, as a result, reactivate more rapidly in response to nutrition post-embryonically. We found that G_0 NSCs primarily occupy dorsal regions of the central nervous system and that G_2 NSCs primarily occupy ventral regions, suggesting that dorsal-ventral patterning cues might influence *p57/dap* expression and consequently the choice between G_0 or G_2 stem cell quiescence. We discovered that the dorsal patterning transcription factor Muscle segment homeobox (Msh, also known as Drop/Dr—Flybase) promotes G_0 quiescence by inducing *p57/dap* expression in a subset of dorsal NSCs. *msh* and *p57/dap* are evolutionarily conserved,

This is an open access article under the CC BY license (http://creativecommons.org/licenses/by/4.0/).

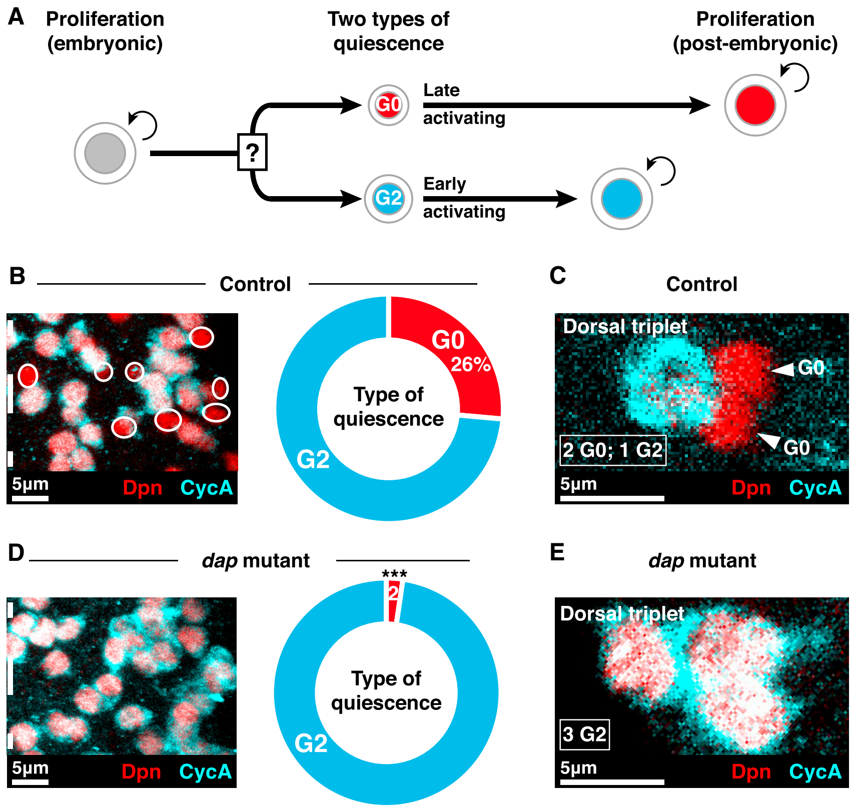

Figure 1. p57/Dap Is Necessary for G0 NSC Quiescence

(A) NSC behaviors during *Drosophila* development. NSCs become quiescent in G_0 (red) or G_2 (cyan) in the late embryo. G_2-quiescent NSCs reactivate before G_0-quiescent NSCs post-embryonically. The factors that determine arrest in G_0 or G_2 are not known.

(B) A single hemi-segment of a control brain. 26% ± 1.0% of NSCs are G_0 quiescent ($CycA^-$; red; circled), and 75% ± 1.0% are G_2 quiescent ($CycA^+$; cyan). Dotted line indicates ventral midline. Maximum intensity projection. n = 10 tVNCs, ~135 NSCs each.

(C) In control brains, two out of three NSCs in the dorsal triplet arrest in G_0 (red; arrowed) and one in G_2 (cyan). Single section image.

(D) A single hemi-segment of a *dap* mutant brain. 2% ± 0.4% of NSCs are G_0 quiescent and 98% ± 0.4% are G_2 quiescent. Maximum intensity projection. n = 10 tVNCs, ~135 NSCs each. The percentage of G_0-quiescent NSCs is significantly different to controls. ***$p = 1.19 \times 10^{-14}$, Student's t test. Dotted line indicates ventral midline.

(E) In *dap* mutant brains, all three NSCs in the dorsal triplet arrest in G_2 (cyan). n = 10 tVNCs. Single section image.

Anterior is up, and dorsal is right in all images.

See also Figure S1.

suggesting that a similar region-specific mechanism might induce different types of stem cell quiescence in the mammalian brain.

RESULTS

Dap Is Necessary for G_0 Quiescence

The factors that regulate the choice between G_0 and G_2 quiescence in NSCs at the end of embryogenesis are not known (Figure 1A). We hypothesized that p57/Dap regulates G_0 stem cell quiescence, as it is the sole *Drosophila* ortholog of the evolutionarily conserved $p21^{CIP}/p27^{KIP1}/p57^{KIP2}$ family of cyclin-dependent kinase inhibitors capable of blocking G_0/G_1>S progression in the cell cycle (de Nooij et al., 1996; Lane et al., 1996). We assessed quiescent NSCs in the loss-of-function mutant dap^{04454} (de Nooij et al., 1996; Spradling et al., 1995) using cyclin A (CycA) expression to distinguish between G_0 ($CycA^-$) and G_2 ($CycA^+$) quiescence, as described previously (Otsuki and Brand, 2018). We focused on the thoracic segments of the ventral nerve cord (tVNC), a region of the central nervous system in which individual NSCs can be identified readily based on spatial position and molecular markers (Lacin and Truman, 2016). Remarkably, we found that G_0-quiescent NSCs had almost completely disappeared in *dap* mutant tVNCs (Figures 1B and 1D). *dap* mutants had an average of 0.6 ± 0.1 G_0 NSCs per hemi-segment, compared to 7.2 ± 0.2 G_0 NSCs per hemi-segment in controls (92% reduction, n = 10 tVNCs, 6 hemi-segments each).

The loss of G_0 NSCs might be due to cell death or premature differentiation. However, we found no change in the total number of NSCs in *dap* mutants (n = 136 ± 2.7 NSCs versus 136 ± 4.0 NSCs, 10 tVNCs each, $p > 0.05$, Student's t test). We hypothesized that the G_0 NSCs might instead have switched to G_2 quiescence. To test this, we focused on quiescent NSCs in the "dorsal triplet," a group of three NSCs (NB2-4, NB2-5, and NB3-5) that can be discriminated unambiguously based on their spatial location in the tVNC (Lacin and Truman, 2016). NB2-4 and NB2-5 in the dorsal triplet normally undergo G_0 quiescence, while NB3-5 arrests in G_2 (Figure 1C) (Otsuki and Brand, 2018). In *dap* mutant brains, we found that NB2-4 and NB2-5 switched from G_0 quiescence to G_2 quiescence, leading to all dorsal triplet NSCs arresting in G_2 (Figure 1E). We observed a similar reduction in G_0-quiescent NSCs and increase in G_2-quiescent NSCs when we knocked down *dap* specifically in NSCs throughout embryogenesis using the *worniu* (*wor*)-GAL4 driver (Figure S1A) (Albertson et al., 2004). We conclude that Dap is necessary for G_0 NSC quiescence and that Dap is required autonomously in NSCs.

We showed previously that G_2 NSCs become activated more rapidly than G_0 NSCs in response to nutrition (Otsuki and Brand, 2018). Therefore, we tested whether *dap* knockdown caused "G_0" NSCs to reactivate at the same rate as G_2 NSCs. We assessed NSC activation at 20 h after larval hatching (ALH), a time point when, in control brains, most G_2 NSCs have reactivated but most G_0 NSCs remain quiescent (Otsuki and Brand, 2018). We found a substantial increase in the number of NSCs that had reactivated by 20 h ALH in *dap* knockdown brains compared to controls (Figure S1B). Thus, the switch from G_0 to G_2 quiescence after *dap* knockdown is sufficient to accelerate the time at which NSCs reactivate.

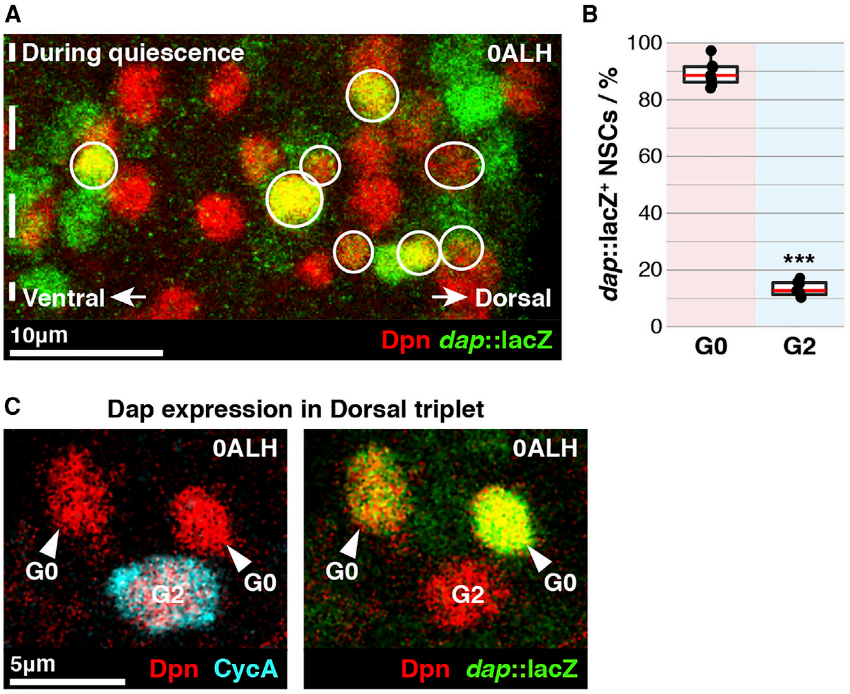

Figure 2. p57/Dap Is Expressed in G_0 NSCs

(A) A single hemi-segment of a tVNC co-stained to visualize NSCs (red) and dap::lacZ (green). *dap*::lacZ$^+$ NSCs are circled. Dotted line indicates ventral midline. Maximum intensity projection. See STAR Methods for explanation of the "dorsal" and "ventral" designations.

(B) Percentages of G_0- and G_2-quiescent NSCs that express *dap::lacZ*. *n* = 7 tVNCs, ~135 NSCs each. ***p = 6.10 × 10^{-14}, Student's t test. Red lines indicate medians.

(C) *dap::lacZ* (green) is expressed in the two G_0 NSCs of the dorsal triplet (arrowed) but not the G_2 NSC.

Anterior is up, and dorsal is right in all images.

See also Figure S2.

G_0 NSCs Express Dap Prior to Quiescence Entry

Next, we assessed the timing of Dap expression in NSCs. The levels of Dap oscillate during the cell cycle (Baumgardt et al., 2014); therefore, to assess Dap transcription, we used a transcriptional reporter in which *lacZ* is inserted at the *dap* locus (hereafter "*dap*::*lacZ*"; Spradling et al., 1999). Importantly, β-galactosidase is stable, and its abundance is not regulated by the cell cycle. We observed a subset of NSCs expressing *dap* prior to quiescence entry (Figure 2A) and, by co-staining for CycA, found that *dap* expression is almost entirely specific to G_0 NSCs. 89% of G_0 NSCs (6.4 ± 0.3 out of 7.2 ± 0.3 per hemi-segment) expressed *dap::lacZ* in contrast to 13% of G_2 NSCs (2.6 ± 0.2 out of 19.7 ± 0.7 per hemi-segment) (Figure 2B). Typifying this pattern, the two G_0 NSCs of the dorsal triplet (NB2-4 and NB2-5) expressed *dap::lacZ* but not the G_2 NSC (NB3-5) (Figure 2C).

Once NSCs enter quiescence, we found that they no longer transcribe or translate Dap (Figures S2A–S2C). Thus, G_0 NSCs express Dap in the embryo but downregulate its expression concomitant with quiescence entry.

G_0- and G_2-Quiescent NSCs Are Distributed in a Dorsal-Ventral Gradient

NSCs acquire their identities through spatial patterning in the developing nervous system. Therefore, the decision to undergo G_0 or G_2 quiescence might be influenced by spatial positioning. By comparing the distributions of G_0 and G_2 NSCs in the tVNC, we noticed a bias toward G_0 NSCs in dorsal regions and G_2 NSCs in ventral regions. In contrast, we found no bias along the anterior-posterior axis (Figures 3A and 3B). This suggested that dorsal-ventral patterning cues might influence *dap* expression and G_0 quiescence entry.

The dorsal-ventral axis of the tVNC is patterned during embryogenesis by three conserved homeobox transcription factors expressed in adjacent columns of the neuroectoderm: *msh* (dorsal identity; human orthologs: MSX1/2/3), *intermediate neuroblasts defective* (*ind*; intermediate identity; human orthologs: GSX1/2), and *ventral nervous system defective* (*vnd*; ventral identity; human orthologs: NKX family) (Chu et al., 1998; D'Alessio and Frasch, 1996; Isshiki et al., 1997; McDonald et al., 1998; Weiss et al., 1998). When NSCs delaminate from the neuroectoderm, they continue to express either Msh, Ind, or Vnd, depending upon their position along the dorso-ventral axis. The only exceptions to this rule are NB3-3, NB3-5, and NB4-4, which delaminate from the Msh$^+$ domain but do not themselves express *msh* (Isshiki et al., 1997) (Figure S3A).

By aligning our G_0/G_2 quiescence map with the Msh/Ind/Vnd expression domains, we found that 5 of 7 Msh$^+$ (dorsal) NSCs per hemi-segment undergo G_0 quiescence, compared to just 1 of 9 Vnd$^+$ (ventral) NSCs (Figures 3C and S3A). We confirmed that most G_0 NSCs originate in the dorsal Msh$^+$ domain by using *msh*-GAL4 (driven by a ~3.5 kb fragment upstream of *msh*) to express GAL4 technique for real-time and clonal expression (G-TRACE) (Evans et al., 2009). We found that an average of 4.2 ± 0.1 out of 7.7 ± 0.2 G_0 NSCs per hemi-segment were Msh > G-TRACE$^+$ (*n* = 8 tVNCs, 6 hemi-segments each) (Figure 3D; compare to 3B). We obtained the same number when we labeled Msh$^+$ NSCs using a *lacZ* insertion at the *msh* locus (Isshiki et al., 1997) (4.2 ± 0.2 out of 7.4 ± 0.4 G_0 NSCs per hemi-segment, *n* = 5 tVNCs, 6 hemi-segments each). Thus, many G_0 NSCs are Msh$^+$ NSCs originating in dorsal regions of the tVNC.

The Dorsal Patterning Factor Msh Promotes G_0 Quiescence

To test whether Msh promotes G_0 quiescence, we quantified G_0- *versus* G_2-quiescent NSCs in *msh* mutant brains at embryonic stage 17. We found that only 3.7 ± 0.1 G_0-quiescent NSCs remained per hemi-segment in $msh^{\Delta 68}$ mutants compared to 6.3 ± 0.3 in controls (41% reduction; *n* = 10 tVNCs, 6 hemi-segments each) (Figure S3B). G_0 NSCs had not died or differentiated in *msh* mutants, as the total number of NSCs was the same as in controls (*n* = 136 ± 3.1 NSCs versus 134 ± 6.2 NSCs, p > 0.05,

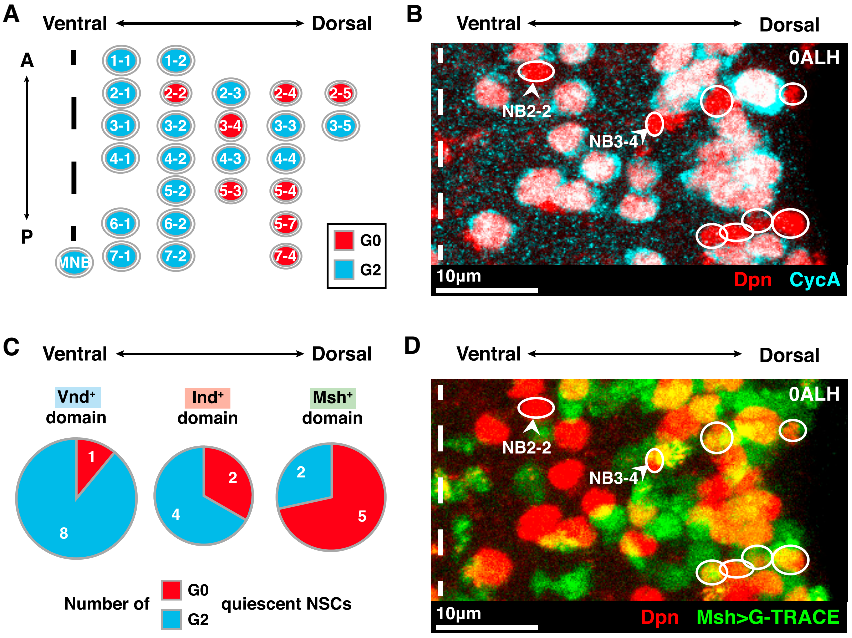

Figure 3. G_0 NSCs Are Prevalent in the Dorsal Nervous System

(A) The distribution of G_0-quiescent NSCs (red) and G_2-quiescent NSCs (cyan) in each hemi-segment of the tVNC. Dotted line indicates ventral midline. G_0 NSCs are prevalent in dorsal regions, and G_2 NSCs in ventral regions. A, anterior; P, posterior. G_0 and G_2 NSCs according to Otsuki and Brand (2018).

(B) A single hemi-segment of a tVNC in the same orientation and colors as in (A). G_0 NSCs are circled. Dotted line indicates ventral midline. To enable comparison with (A), the G_0 NSCs NB2-2 and NB3-4 are indicated. Maximum intensity projection.

(C) Comparison of the numbers of G_0- *versus* G_2-quiescent NSCs per hemi-segment in ventral (Vnd^+), intermediate (Ind^+), and dorsal (Msh^+) regions. Data assembled using data from Chu et al. (1998); D'Alessio and Frasch (1996); Isshiki et al. (1997); McDonald et al. (1998); and Weiss et al. (1998).

(D) The same tVNC hemi-segment as in (B), with Msh-expressing regions labeled in green using G-TRACE. G_0 NSCs are circled. Most G_0 NSCs reside in the Msh^+ domain. To enable comparison with (A), the G_0 NSCs NB2-2 and NB3-4 (that do not express Msh) are indicated. Maximum intensity projection.

Anterior is up, and dorsal is right in all images.

See also Figure S3.

Welch's test). This indicated that a subset of NSCs switches from G_0 to G_2 quiescence in *msh* mutants, as occurs in *dap* mutants.

As Msh is necessary for dorsal patterning (Isshiki et al., 1997), we surmised that dorsal (but not ventral) NSCs switch from G_0 to G_2 quiescence in *msh* mutants. To test this, we focused again on dorsal triplet NSCs, as they are three of the most dorsally located NSCs in the tVNC (Lacin and Truman, 2016). The two G_0 NSCs in the dorsal triplet (NB2-4 and NB2-5) express *msh*, whereas the G_2 NSC (NB3-5) does not (Figure 4A). In *msh* mutant brains, we found that NB2-4 and NB2-5 switched from G_0 quiescence to G_2 quiescence, resulting in all three dorsal triplet NSCs becoming quiescent in G_2 (Figure 4B). Thus, Msh promotes G_0 quiescence in dorsal NSCs. As a comparison, we assessed a G_0 NSC on the ventral side of the tVNC (NB2-2), which never expresses Msh normally. As expected, NB2-2 remained G_0 arrested in *msh* mutants (Figure S3C). Thus, Msh promotes G_0 quiescence in dorsal, but not ventral, NSCs.

We tested whether the ventral patterning factor Vnd also has the ability to promote G_0 quiescence in the tVNC. NB2-2 is the only Vnd^+ NSC per hemi-segment that undergoes G_0 quiescence (Figure S3A). In vnd^6 mutants, we found that NB2-2 did not switch from G_0 to G_2 quiescence (Figures S3D and S3E′). We conclude that Vnd does not promote G_0 quiescence ventrally.

Msh Directly Induces *Dap* Expression in Dorsal NSCs

Dap and Msh both promote G_0 stem cell quiescence and could act in a linear pathway. We found that the same NSCs (for example, NB2-4 and NB2-5) co-express Msh and Dap during embryogenesis (Figures 2C and 4A). We tested whether Msh induces *dap* expression, as NSCs begin to express *msh* from embryonic stage 9/10 while *dap* expression initiates later, from embryonic stage 11 (Baumgardt et al., 2014; de Nooij et al., 1996; Isshiki et al., 1997; Lane et al., 1996). Consistent with Msh promoting *dap* expression, we found that dorsal NSCs lost Dap expression in *msh* mutant embryos (Figures S4A and S4B). Only 2.6 ± 0.3 NSCs per hemi-segment expressed Dap in *msh* mutant embryos compared to 4.9 ± 0.2 NSCs in controls, a decrease of almost 50% (Figure 4C). Thus, Msh is one upstream regulator that promotes Dap expression in the central nervous system.

Msh might promote Dap expression in NSCs directly, by binding to the *dap* locus and inducing transcription, or indirectly. The genomic targets of Msh are not known. We therefore elucidated the genome-wide binding targets of Msh in NSCs *in vivo* using Targeted DamID (TaDa) (Marshall and Brand, 2015; Marshall et al., 2016; Southall et al., 2013). We generated transgenic *Drosophila* carrying UAST-LT3-NDam-Msh, which we expressed specifically in NSCs *in vivo* using *wor*-GAL4. We found that Msh binds directly to the *dap* locus in NSCs (Figures 4D and S4C). Remarkably, Msh binding at the *dap* locus precisely matched the enhancer sequences previously shown to be sufficient for *dap* expression in the embryonic central nervous system (Liu et al., 2002; Figure 4D).

We conclude that the dorsal patterning factor Msh binds directly to *dap* enhancers in dorsal NSCs and induces *dap* expression. As Dap promotes G_0 quiescence, this leads to a preferential distribution of G_0-quiescent NSCs in dorsal regions of the brain (Figure 4E). In contrast, Msh is not expressed in ventral regions, where more NSCs undergo G_2 quiescence (Figure 4E). G_2-quiescent NSCs become activated first, followed by G_0-quiescent NSCs, during larval life (Otsuki and Brand, 2018). Thus, differential *dap* expression results directly in functional heterogeneity among quiescent stem cells.

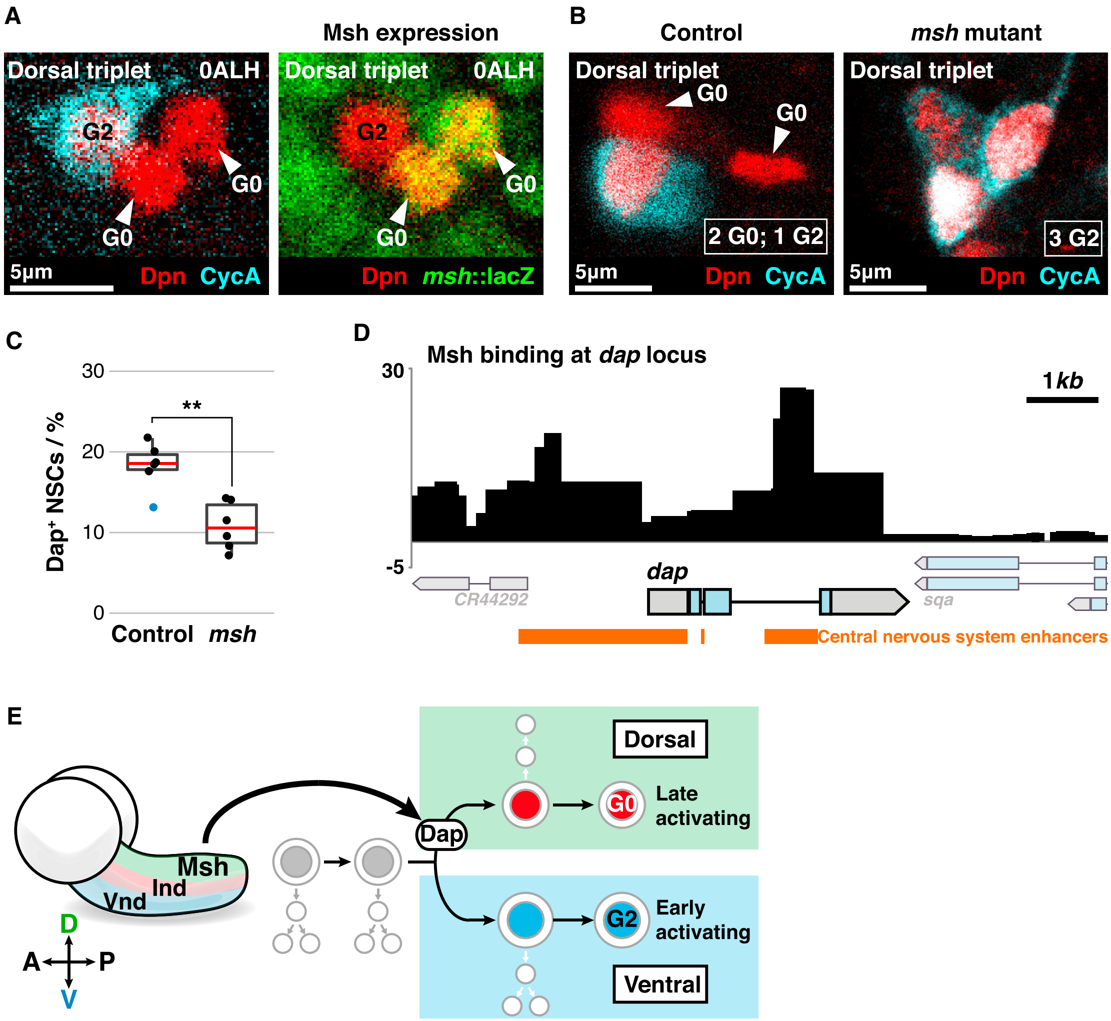

Figure 4. Msh Induces G_0 Quiescence by Directly Promoting p57/Dap Expression

(A) *msh*::lacZ (green) is expressed in both G_0 NSCs (arrowed) of the dorsal triplet but not in the G_2 NSC.

(B) In *msh* mutants, all NSCs in the dorsal triplet arrest in G_2 quiescence (cyan). Arrows indicate G_0 NSCs in controls.

(C) Quantification of Dap⁺ NSCs in control ($msh^{\Delta68}$ heterozygous) versus $msh^{\Delta68}$ mutant brains at embryonic stage 13. Dap expression was assessed using anti-Dap antiserum. $n = 6$ tVNCs/genotype, ~140 NSCs each. **$p = 1.36 \times 10^{-3}$, Student's t test. Red lines indicate medians. Blue data point is an outlier.

(D) Msh binding at the *dap* locus, assessed specifically in embryonic NSCs using TaDa (Southall et al., 2013). Unlogged data assembled from three biological replicates. Orange bars indicate enhancers that were characterized functionally to drive *dap* expression in the central nervous system (Liu et al., 2002).

(E) Model for dorsal-ventral control of stem cell quiescence. The dorsal patterning factor Msh directly induces *dap* expression in dorsal NSCs, causing them to undergo the type 1 > 0 proliferation switch, followed by arrest in G_0 quiescence. Ventral NSCs do not express Msh or Dap, do not undergo type 0 proliferation, and arrest in G_2 quiescence.

Anterior is up, and dorsal is right in all images.

See also Figures S3 and S4.

DISCUSSION

Quiescent NSCs in the mammalian brain exhibit significant heterogeneity in function and molecular profile (Dulken et al., 2017; Llorens-Bobadilla et al., 2015; Shin et al., 2015). In order to harness quiescent NSCs for regenerative therapies, it will be necessary to identify the regulators that control different types of stem cell quiescence. Here, we have identified Msh-p57/Dap as one regulatory arc that induces G_0 stem cell quiescence in dorsal NSCs of the central nervous system. Together with our previous finding that the pseudokinase Tribbles (Trbl) regulates G_2 NSCs (Otsuki and Brand, 2018), we have elucidated the mechanisms that allocate and regulate NSCs entering G_0 or G_2 quiescence.

We found that Dap expression (1) initiates in a subset of dividing NSCs during embryogenesis and (2) induces these NSCs to enter G_0 quiescence. These features show remarkable parallels with p57 expression and function in mammalian NSC quiescence. In the developing mouse brain, p57 expression increases in a subset of proliferating embryonic NSCs around

E15.5, inducing them to enter G_0 quiescence (Furutachi et al., 2015). Once quiescent, p57-expressing NSCs are retained into the adult V-SVZ (Fuentealba et al., 2015; Furutachi et al., 2015). Intriguingly, p57 deletion in the developing mouse brain was shown to reduce, but not eliminate, the emergence of quiescent NSCs in the adult V-SVZ, suggesting that some NSCs in the mammalian brain do not require p57 for quiescence entry (Furutachi et al., 2015). We found that ~75% of NSCs do not express Dap in the *Drosophila* brain and that these NSCs later become quiescent in G_2 instead of G_0. The *p57*-independent NSCs in the mouse brain might be comparable to G_2-quiescent NSCs in *Drosophila*.

In both mammals and *Drosophila*, NSCs are set aside to become quiescent during proliferative stages. Interestingly, Dap has been shown to induce a switch in NSC proliferation mode during *Drosophila* embryogenesis (Baumgardt et al., 2014). At cell division, most NSCs in the brain produce a ganglion mother cell (GMC) that divides once to generate two neurons and/or glia (type 1 proliferation). During mid-embryogenesis, Dap-expressing NSCs switch from type 1 proliferation to type 0 proliferation, in which the GMC differentiates directly into a post-mitotic cell without dividing (Baumgardt et al., 2009, 2014; Karcavich and Doe, 2005). It has been suggested that all NSCs express Dap during embryogenesis (Baumgardt et al., 2014). We now show that only a subset of embryonic NSCs, the G_0 population, expresses Dap. We propose that Dap-expressing NSCs first switch from type 1 to type 0 proliferation at mid-embryogenesis, before undergoing G_0 quiescence (Figure 4E). In contrast, Dap non-expressing NSCs remain in type 1 proliferation mode and undergo G_2 quiescence (Figure 4E). A similar switch in NSC proliferation mode may also precede quiescence entry in the developing mammalian brain.

We have shown that the dorsal patterning factor Msh directly induces Dap expression in dorsal NSCs, causing them to enter G_0 quiescence. The switch from G_0 to G_2 quiescence in *msh* mutants is less severe than in *dap* mutants, suggesting that Msh is one of the several regulators upstream of Dap expression. Indeed, Hox genes, Notch signaling, and temporal patterning factors have been shown to influence Dap expression in the brain (Baumgardt et al., 2014; Bivik et al., 2016; Monedero Cobeta et al., 2017; Gunnar et al., 2016).

The distribution of G_0- versus G_2-quiescent NSCs along the dorsal-ventral brain axis is striking, given that G_2-quiescent NSCs reactivate to produce neurons faster than G_0 NSCs (Otsuki and Brand, 2018). It is possible that ventral neurons must be generated first to direct dorsal neurons to form the correct neural circuits, in a role comparable to the pioneer neurons of the embryonic nervous system (Jacobs and Goodman, 1989). The dorsal-ventral patterning system is well conserved evolutionarily, and the homologs of Msh, Ind, and Vnd are also expressed in columns during mammalian brain development (Urbach and Technau, 2008). It is not known whether dorsal-ventral patterning controls *p57* expression in NSCs in the mammalian brain. However, MSX1, one of the Msh homologs, binds upstream of the *p57* locus in cultured mouse myoblasts (Wang et al., 2011). Our finding that Msh directly induces *dap* expression in NSCs raises the possibility that dorsal-ventral control of p57/Dap and NSC quiescence is conserved in the mammalian brain.

STAR★METHODS

Detailed methods are provided in the online version of this paper and include the following:

- KEY RESOURCES TABLE
- CONTACT FOR REAGENT AND RESOURCE SHARING
- EXPERIMENTAL MODEL AND SUBJECT DETAILS
  - *Drosophila melanogaster* Rearing and Genetics
- METHOD DETAILS
  - Antibody Staining
  - Designation of 'Dorsal' and 'Ventral' in Confocal Microscopy Images
  - *dap* Mutant Analysis
  - *dap* Knockdown in NSCs
  - *msh*>G-TRACE
  - Generation of UASTattB-LT3-NDam-Msh Flies for TaDa
  - Identification of Msh Genome-Wide Binding Targets Using TaDa
  - Image Acquisition and Processing
- QUANTIFICATION AND STATISTICAL ANALYSIS

SUPPLEMENTAL INFORMATION

Supplemental Information can be found with this article online at https://doi.org/10.1016/j.devcel.2019.02.015.

ACKNOWLEDGMENTS

We thank F. Jiménez Díaz-Benjumea (Centro de Biología Molecular Severo Ochoa, Spain), Y. Kimata (University of Cambridge, UK), C. Lehner (University of Zurich, Switzerland), the Asian Distribution Centre for Segmentation Antibodies, the Bloomington Drosophila Stock Centre (BDSC), and Kyoto Drosophila Stock Centre for reagents. We thank R. Yakob for plasmid injections to generate transgenic UAST-LT3-NDam-Msh flies, C.M. Davidson for performing the TaDa protocol, and R. Krautz for analyzing the Msh TaDa binding data. This work was funded by the Royal Society Darwin Trust Research Professorship and Wellcome Trust Senior Investigator Award 103792 to A.H.B. and Wellcome Trust PhD Studentship 097423 to L.O. A.H.B. acknowledges core funding to the Gurdon Institute from the Wellcome Trust (092096) and The Royal Society, United Kingdom (C6946/A14492).

AUTHOR CONTRIBUTIONS

L.O. and A.H.B. designed the experiments, analyzed the data, and wrote the paper. L.O. performed the experiments.

DECLARATION OF INTERESTS

The authors declare no competing interests.

Received: March 16, 2018
Revised: January 28, 2019
Accepted: February 15, 2019
Published: March 21, 2019

REFERENCES

Albertson, R., Chabu, C., Sheehan, A., and Doe, C.Q. (2004). Scribble protein domain mapping reveals a multistep localization mechanism and domains necessary for establishing cortical polarity. J. Cell. Sci. *117*, 6061–6070.

Baumgardt, M., Karlsson, D., Salmani, B.Y., Bivik, C., MacDonald, R.B., Gunnar, E., and Thor, S. (2014). Global programmed switch in neural daughter

cell proliferation mode triggered by a temporal gene cascade. Dev. Cell *30*, 192–208.

Baumgardt, M., Karlsson, D., Terriente, J., Díaz-Benjumea, F.J., and Thor, S. (2009). Neuronal subtype specification within a lineage by opposing temporal feed-forward loops. Cell *139*, 969–982.

Bivik, C., MacDonald, R.B., Gunnar, E., Mazouni, K., Schweisguth, F., and Thor, S. (2016). Control of neural daughter cell proliferation by multi-level Notch/Su(H)/E(spl)-HLH signaling. PLoS Genet. *12*, e1005984.

Campos-Ortega, J.A., and Hartenstein, V. (1985). The Embryonic Origin of Drosophila melanogaster (Berlin, Germany: Springer-Verlag), p. 227.

Caygill, E.E., and Brand, A.H. (2017). miR-7 buffers differentiation in the developing Drosophila visual system. Cell Rep. *20*, 1255–1261.

Chaker, Z., Codega, P., and Doetsch, F. (2016). A mosaic world: puzzles revealed by adult neural stem cell heterogeneity. Wires Dev. Biol. *5*, 640–658.

Cheung, T.H., and Rando, T.A. (2013). Molecular regulation of stem cell quiescence. Nat. Rev. Mol. Cell Biol. *14*, 329–340.

Chu, H., Parras, C., White, K., and Jiménez, F. (1998). Formation and specification of ventral neuroblasts is controlled by vnd in Drosophila neurogenesis. Genes Dev. *12*, 3613–3624.

Monedero Cobeta, I.M., Salmani, B.Y., and Thor, S. (2017). Anterior-posterior gradient in neural stem and daughter cell proliferation governed by spatial and temporal hox control. Curr. Biol. *27*, 1161–1172.

D'Alessio, M., and Frasch, M. (1996). Msh may play a conserved role in dorsoventral patterning of the neuroectoderm and mesoderm. Mech. Dev. *58*, 217–231.

de Nooij, J.C., Letendre, M.A., and Hariharan, I.K. (1996). A cyclin-dependent kinase inhibitor, dacapo, is necessary for timely exit from the cell cycle during Drosophila embryogenesis. Cell *87*, 1237–1247.

Doetsch, F., García-Verdugo, J.M., and Alvarez-Buylla, A. (1999). Regeneration of a germinal layer in the adult mammalian brain. Proc. Natl. Acad. Sci. USA *96*, 11619–11624.

Dulken, B.W., Leeman, D.S., Boutet, S.C., Hebestreit, K., and Brunet, A. (2017). Single-cell transcriptomic analysis defines heterogeneity and transcriptional dynamics in the adult neural stem cell lineage. Cell Rep. *18*, 777–790.

Encinas, J.M., and Fitzsimons, C.P. (2017). Gene regulation in adult neural stem cells. Current challenges and possible applications. Adv. Drug Deliv. Rev. *120*, 118–132.

Evans, C.J., Olson, J.M., Ngo, K.T., Kim, E., Lee, N.E., Kuoy, E., Patananan, A.N., Sitz, D., Tran, P., Do, M.T., et al. (2009). G-TRACE: rapid Gal4-based cell lineage analysis in Drosophila. Nat. Methods *6*, 603–605.

Fuentealba, L.C., Rompani, S.B., Parraguez, J.I., Obernier, K., Romero, R., Cepko, C.L., and Alvarez-Buylla, A. (2015). Embryonic origin of postnatal neural stem cells. Cell *161*, 1644–1655.

Furutachi, S., Miya, H., Watanabe, T., Kawai, H., Yamasaki, N., Harada, Y., Imayoshi, I., Nelson, M., Nakayama, K.I., Hirabayashi, Y., et al. (2015). Slowly dividing neural progenitors are an embryonic origin of adult neural stem cells. Nat. Neurosci. *18*, 657–665.

Gunnar, E., Bivik, C., Starkenberg, A., and Thor, S. (2016). Sequoia controls the type I>0 daughter proliferation switch in the developing Drosophila nervous system. Development *143*, 3774–3784.

Hoskins, R.A., Carlson, J.W., Wan, K.H., Park, S., Mendez, I., Galle, S.E., Booth, B.W., Pfeiffer, B.D., George, R.A., Svirskas, R., et al. (2015). The Release 6 reference sequence of the *Drosophila melanogaster* genome. Genome Res. *25*, 445–458.

Isshiki, T., Takeichi, M., and Nose, A. (1997). The role of the msh homeobox gene during Drosophila neurogenesis: implication for the dorsoventral specification of the neuroectoderm. Development *124*, 3099–3109.

Jacobs, J.R., and Goodman, C.S. (1989). Embryonic development of axon pathways in the Drosophila CNS. II. Behavior of pioneer growth cones. J. Neurosci. *9*, 2412–2422.

Jhaveri, D.J., O'Keeffe, I., Robinson, G.J., Zhao, Q.Y., Zhang, Z.H., Nink, V., Narayanan, R.K., Osborne, G.W., Wray, N.R., and Bartlett, P.F. (2015). Purification of neural precursor cells reveals the presence of distinct, stimulus-specific subpopulations of quiescent precursors in the adult mouse hippocampus. J. Neurosci. *35*, 8132–8144.

Jiménez, F., Martin-Morris, L.E., Velasco, L., Chu, H., Sierra, J., Rosen, D.R., and White, K. (1995). vnd, a gene required for early neurogenesis of Drosophila, encodes a homeodomain protein. EMBO J. *14*, 3487–3495.

Karcavich, R., and Doe, C.Q. (2005). Drosophila neuroblast 7-3 cell lineage: a model system for studying programmed cell death, Notch/Numb signaling, and sequential specification of ganglion mother cell identity. J. Comp. Neurol. *481*, 240–251.

Kosman, D., Small, S., and Reinitz, J. (1998). Rapid preparation of a panel of polyclonal antibodies to Drosophila segmentation proteins. Dev. Genes Evol. *208*, 290–294.

Lacin, H., and Truman, J.W. (2016). Lineage mapping identifies molecular and architectural similarities between the larval and adult Drosophila central nervous system. Elife *5*, e13399.

Lane, M.E., Sauer, K., Wallace, K., Jan, Y.N., Lehner, C.F., and Vaessin, H. (1996). Dacapo, a cyclin-dependent kinase inhibitor, stops cell proliferation during Drosophila development. Cell *87*, 1225–1235.

Liu, T.H., Li, L., and Vaessin, H. (2002). Transcription of the Drosophila CKI gene dacapo is regulated by a modular array of cis-regulatory sequences. Mech. Dev. *112*, 25–36.

Llorens-Bobadilla, E., Zhao, S., Baser, A., Saiz-Castro, G., Zwadlo, K., and Martin-Villalba, A. (2015). Single-cell transcriptomics reveals a population of dormant neural stem cells that become activated upon brain injury. Cell Stem Cell *17*, 329–340.

Lugert, S., Basak, O., Knuckles, P., Haussler, U., Fabel, K., Götz, M., Haas, C.A., Kempermann, G., Taylor, V., and Giachino, C. (2010). Quiescent and active hippocampal neural stem cells with distinct morphologies respond selectively to physiological and pathological stimuli and aging. Cell Stem Cell *6*, 445–456.

Marshall, O.J., and Brand, A.H. (2015). damidseq_pipeline: an automated pipeline for processing DamID sequencing datasets. Bioinformatics *31*, 3371–3373.

Marshall, O.J., Southall, T.D., Cheetham, S.W., and Brand, A.H. (2016). Cell-type-specific profiling of protein-DNA interactions without cell isolation using targeted DamID with next-generation sequencing. Nat. Protoc. *11*, 1586–1598.

McDonald, J.A., Holbrook, S., Isshiki, T., Weiss, J., Doe, C.Q., and Mellerick, D.M. (1998). Dorsoventral patterning in the Drosophila central nervous system: the vnd homeobox gene specifies ventral column identity. Genes Dev. *12*, 3603–3612.

Otsuki, L., and Brand, A.H. (2018). Cell cycle heterogeneity directs the timing of neural stem cell activation from quiescence. Science *360*, 99–102.

Robinson, J.T., Thorvaldsdóttir, H., Winckler, W., Guttman, M., Lander, E.S., Getz, G., and Mesirov, J.P. (2011). Integrative genomics viewer. Nat. Biotechnol. *29*, 24–26.

Schindelin, J., Arganda-Carreras, I., Frise, E., Kaynig, V., Longair, M., Pietzsch, T., Preibisch, S., Rueden, C., Saalfeld, S., Schmid, B., et al. (2012). Fiji: an open-source platform for biological-image analysis. Nat. Methods *9*, 676–682.

Seri, B., García-Verdugo, J.M., McEwen, B.S., and Alvarez-Buylla, A. (2001). Astrocytes give rise to new neurons in the adult mammalian hippocampus. J. Neurosci. *21*, 7153–7160.

Shin, J., Berg, D.A., Zhu, Y., Shin, J.Y., Song, J., Bonaguidi, M.A., Enikolopov, G., Nauen, D.W., Christian, K.M., Ming, G.L., et al. (2015). Single-cell RNA-seq with waterfall reveals molecular cascades underlying adult neurogenesis. Cell Stem Cell *17*, 360–372.

Southall, T.D., Gold, K.S., Egger, B., Davidson, C.M., Caygill, E.E., Marshall, O.J., and Brand, A.H. (2013). Cell-type-specific profiling of gene expression and chromatin binding without cell isolation: assaying RNA Pol II occupancy in neural stem cells. Dev. Cell *26*, 101–112.

Spradling, A.C., Stern, D., Beaton, A., Rhem, E.J., Laverty, T., Mozden, N., Misra, S., and Rubin, G.M. (1999). The Berkeley Drosophila Genome Project

gene disruption project: single P-element insertions mutating 25% of vital Drosophila genes. Genetics *153*, 135–177.

Spradling, A.C., Stern, D.M., Kiss, I., Roote, J., Laverty, T., and Rubin, G.M. (1995). Gene disruptions using P transposable elements: an integral component of the Drosophila genome project. Proc. Natl. Acad. Sci. USA *92*, 10824–10830.

Urbach, R., and Technau, G.M. (2008). Dorsoventral patterning of the brain: a comparative approach. Adv. Exp. Med. Biol. *628*, 42–56.

Urbán, N., van den Berg, D.L.C., Forget, A., Andersen, J., Demmers, J.A.A., Hunt, C., Ayrault, O., and Guillemot, F. (2016). Return to quiescence of mouse neural stem cells by degradation of a proactivation protein. Science *353*, 292–295.

Wang, J., Kumar, R.M., Biggs, V.J., Lee, H., Chen, Y., Kagey, M.H., Young, R.A., and Abate-Shen, C. (2011). The Msx1 homeoprotein recruits Polycomb to the nuclear periphery during development. Dev. Cell *21*, 575–588.

Weiss, J.B., Von Ohlen, T., Mellerick, D.M., Dressler, G., Doe, C.Q., and Scott, M.P. (1998). Dorsoventral patterning in the Drosophila central nervous system: the intermediate neuroblasts defective homeobox gene specifies intermediate column identity. Genes Dev. *12*, 3591–3602.

Whitfield, W.G., Gonzalez, C., Maldonado-Codina, G., and Glover, D.M. (1990). The A- and B-type cyclins of Drosophila are accumulated and destroyed in temporally distinct events that define separable phases of the G2-M transition. EMBO J. *9*, 2563–2572.

STAR★METHODS

KEY RESOURCES TABLE

REAGENT or RESOURCE	SOURCE	IDENTIFIER
**Antibodies**		
Chicken polyclonal anti-β-galactosidase	abcam	Cat# ab9361, RRID:AB_307210
Rabbit polyclonal anti-Cyclin A	Whitfield et al., 1990	ID: rb270
Rabbit polyclonal anti-Dacapo	C Lehner (University of Zurich, Switzerland)	N/A
Guinea pig anti-Deadpan	Caygill and Brand, 2017	N/A
Rat anti-Deadpan	abcam	Cat# ab195173, RRID:AB_2687586
Guinea pig anti-Runt	Kosman et al., 1998	#638
Rat anti-Worniu	abcam	Cat# ab196362
**Deposited Data**		
D. melanogaster Release 6 Genome assembly	Berkeley Drosophila Genome Project; Hoskins et al., 2015	https://www.ncbi.nlm.nih.gov/assembly/GCF_000001215.4
**Experimental Models: Organisms/Strains**		
D. melanogaster: w^{1118}	Bloomington Drosophila Stock Centre	BDSC Cat# 3605, RRID:BDSC_3605
D. melanogaster: P{PZ}dap04454	Bloomington Drosophila Stock Centre	BDSC Cat# 11377, RRID:BDSC_11377
D. melanogaster: Df(2R)Exel9016	Bloomington Drosophila Stock Centre	BDSC Cat# 7867, RRID:BDSC_7867
D. melanogaster: P{w^{+mC}=lacW}dap^{k07309}	Bloomington Drosophila Stock Centre	BDSC Cat# 10406, RRID:BDSC_10406
D. melanogaster: P{w^{+mC}=wor.GAL4.A}2	Bloomington Drosophila Stock Centre	BDSC Cat# 56553, RRID:BDSC_56553
D. melanogaster: P{y$^{+t7.7}$ v+^{t1}.^{8}= TRiP.HMS05362}attP40	Bloomington Drosophila Stock Centre	BDSC Cat# 64026, RRID:BDSC_64026
D. melanogaster: P{y$^{+t7.7}$ v$^{+t1.8}$= VALIUM20-mCherry}attP2	Bloomington Drosophila Stock Centre	BDSC Cat# 35785, RRID:BDSC_35785
D. melanogaster: P{y$^{+t7.7}$ w^{+mC}= GMR19B03-GAL4}attP2	Bloomington Drosophila Stock Centre	BDSC Cat# 49830, RRID:BDSC_49830
D. melanogaster: P{w^{+mC}=UAS-RedStinger}4, P{w^{+mC}=UAS-FLP.D}JD1, P{w^{+mC}= Ubi-p63E(FRT.STOP)Stinger}9F6	Bloomington Drosophila Stock Centre	BDSC Cat# 28280, RRID:BDSC_28280
D. melanogaster: msh[delta68]	Kyoto DGGR	Kyoto Cat# 116970
D. melanogaster: msh[delta89-lacZ]	Kyoto DGGR	Kyoto Cat# 116971
D. melanogaster: vnd^{6}	F J Díaz-Benjumea (Centro de Biología Molecular Severo Ochoa, Spain)	N/A
D. melanogaster: UAST-LT3-NDam	Southall et al., 2013	N/A
D. melanogaster: UAST-LT3-NDam-Msh	This paper	N/A
**Oligonucleotides**		
Primer: Dam-Msh-FWD-XhoI TCATCTCGAGATGTTAAAGCTCAGCCCAGC	This paper	N/A
Primer: Dam-Msh-REV-XbaI TCATTCTAGATTATCCCAGGTGCATCAGGC	This paper	N/A
**Recombinant DNA**		
Plasmid: pUASTattB-LT3-NDam	Southall et al., 2013	N/A
Plasmid: pUASTattB-LT3-NDam-Msh	This paper	N/A
cDNA: clone LD04235 (*msh*)	*Drosophila* Genomics Resource Centre	DGRC Cat# 4281

CONTACT FOR REAGENT AND RESOURCE SHARING

Further information and requests for resources and reagents should be directed to and will be fulfilled by the Lead Contact, Andrea H. Brand (a.brand@gurdon.cam.ac.uk).

EXPERIMENTAL MODEL AND SUBJECT DETAILS

Drosophila melanogaster Rearing and Genetics

Drosophila melanogaster were reared at 25°C except for RNAi experiments, which were conducted at 29°C. Embryos were collected onto yeasted apple juice plates and staged according to (Campos-Ortega and Hartenstein, 1985). For larval experiments, larvae were transferred to a fresh, yeasted food plate within one hour of hatching (designated 0 hours after larval hatching (ALH)) and allowed to develop to the required stage. The following stocks were obtained from the Bloomington Drosophila Stock Centre: w^{1118}, dap^{04454} (BL11377) (de Nooij et al., 1996; Spradling et al., 1995), dap^{Df9016} (BL7867), dap^{k07309} (*dap::lacZ*; BL10406) (Spradling et al., 1999), *wor*-GAL4 (Albertson et al., 2004), P{TRiP.HMS05362}attP40 (*dap* RNAi; BL64026), P{GMR19B03-GAL4}attP2 (*msh*-GAL4; BL49830), G-TRACE (BL28280) (Evans et al., 2009), P{VALIUM20-mCherry}attP2 (*mCherry* RNAi; BL35785). The following stocks were obtained from the Kyoto *Drosophila* Stock Centre: $msh^{\Delta 68}$ (116970) (Isshiki et al., 1997), $msh^{lacZ\text{-}\Delta 89}$ (*msh::lacZ*; 116971) (Isshiki et al., 1997). vnd^{6} was a kind gift from Fernando Jiménez Díaz-Benjumea (Centro de Biología Molecular Severo Ochoa, Spain) (Jiménez et al., 1995). UAST-LT3-NDam was published previously (Southall et al., 2013). The following stock was generated for this study: UAST-LT3-NDam-Msh.

METHOD DETAILS

Antibody Staining

Embryos were washed into a nitex basket with water, dechorionated for 3 minutes in 50% bleach/water, then fixed for 20 minutes in 4% formaldehyde (in PBS)/heptane on a rolling shaker. Fixed embryos were stored in methanol at -20°C until use. For immunostaining: fixed embryos were re-hydrated in PBTx (0.3% Triton X-100/PBS), blocked for 15 minutes in 10% normal goat serum/PBTx, then incubated overnight with primary antibodies at 4°C. Primary antibodies were washed off with PBTx and replaced with secondary antibodies for 2 hours at room temperature, or overnight at 4°C. Secondary antibodies were washed off with PBTx and embryos were mounted in 50% glycerol/PBS.

Larval brains were dissected in PBS, then fixed for 20 minutes in 4% formaldehyde/PBS. Fixed brains were washed three times in PBTx, blocked for 15 minutes in 10% normal goat serum/PBTx, then immunostained as described for embryos. Larval brains were mounted in Vectashield mounting medium (Vector Laboratories).

The following primary antisera were used, diluted in PBTx: chicken anti-βgal 1:1,000 (abcam, ab9361), rabbit anti-CycA 1:500 ((Whitfield et al., 1990), rb270), rabbit anti-Dap 1:600 (gift from C. Lehner), guinea pig anti-Dpn 1:5,000 (Caygill and Brand, 2017), rat anti-Dpn 1:100 (abcam, 11D1BC7, ab195173), guinea pig anti-Run 1:200 (Kosman et al., 1998, 638), rat anti-Wor 1:100 (abcam, 5A3AD2, ab196362). Primary antibodies were detected using Alexa Fluor-conjugated secondary antibodies (Thermo Fisher Scientific) diluted 1:500 in PBTx.

Designation of ‘Dorsal’ and ‘Ventral’ in Confocal Microscopy Images

tVNCs were imaged in ventral view. The left-right axis in each image corresponds to medial-lateral in the tVNC. During *Drosophila* embryogenesis, ventral NSCs delaminate medially and dorsal NSCs delaminate laterally. Thus, the medial-lateral axis is labelled dorsal-ventral in confocal images.

dap Mutant Analysis

The loss of function allele dap^{04454} was crossed to the genomic deficiency dap^{Df9016} and designated ‘*dap* mutant’ throughout this study, as described in (Baumgardt et al., 2014).

dap Knockdown in NSCs

dap RNAi was expressed in NSCs using *wor*-GAL4. Flies were raised at 29°C and assessed at 0ALH. To assess stem cell activation, dap RNAi (or control) larvae were transferred within 1 hour ALH to a new food plate and kept for a further 20 hours at 25°C. Brains were dissected at 20ALH, and co-stained for Dpn (to label NSCs) and Wor (to label activated NSCs).

msh>G-TRACE

G-TRACE was expressed in Msh-expressing NSCs using GMR19B03-GAL4. Flies were raised at 25°C and assessed at 0ALH. The image in Figure 3D depicts the ‘historical expression’ reporter from the G-TRACE cassette.

Generation of UASTattB-LT3-NDam-Msh Flies for TaDa

Full length *msh* cDNA was PCR amplified from Clone LD04235 (DGRC Bloomington ID #4281), with flanking XhoI and XbaI restriction sites, using the following primers:

Dam-Msh-FWD-XhoI
5’-TCATCTCGAGATGTTAAAGCTCAGCCCAGC-3’
Dam-Msh-REV-XbaI
5’-TCATTCTAGATTATCCCAGGTGCATCAGGC-3’

The amplified PCR product was digested with XhoI and XbaI enzymes and ligated into pUASTattB-LT3-NDam (Southall et al., 2013), also digested with XhoI and XbaI, to generate pUASTattB-LT3-NDam-Msh.

Stable transgenic flies (UAST-LT3-NDam-Msh) were established by injecting pUASTattB-LT3-NDam-Msh into embryos expressing phiC31 integrase and carrying the attP2 genomic landing site on III. Successful transgenesis was confirmed by sequencing.

Identification of Msh Genome-Wide Binding Targets Using TaDa

Msh targets were identified using TaDa (Southall et al., 2013). *wor*-GAL4 flies were crossed to control (UAST-LT3-NDam) or test (UAST-LT3-NDam-Msh) flies. Embryos were collected onto apple juice plates for a two-hour period and developed at 25°C for 20 hours. Embryos were washed into a nitex basket using distilled water and dechorionated by swirling for three minutes in 50% bleach. Dechorionated embryos were washed well with distilled water, transferred to an Eppendorf tube, liquid removed and frozen at -80°C until ready for use. Three replicate experiments were conducted and ~25μl of embryos were used for each replicate.

Genomic DNA was extracted from embryos using the QiaAmp DNA micro kit (Qiagen), as described previously in the TaDa protocol (Marshall et al., 2016). In brief, frozen embryos were re-suspended in PBS containing 110mM EDTA and 0.25μg of RNase A, then disrupted mechanically using an electric drill. 20μl of Proteinase K (QiaAmp DNA micro kit) were added, and the sample left for 1 minute at room temperature. 200μl of Buffer AL were added, the tube was inverted gently to mix and then incubated at 56°C overnight. The following day, the sample was cooled to room temperature and 200μl of 100% ethanol added. The sample was applied to a QiaAmp DNA micro kit spin column, then washed and centrifuged on the column with AW1 followed by AW2 solution. The column was transferred to a clean tube and centrifuged again to dry. Finally, the column was transferred to a clean tube and the genomic DNA eluted in 50μl of AE buffer. Genomic DNA was digested with DpnI enzyme (NEB) overnight at 37°C, ligated with DamID adaptors, then digested with DpnII enzyme. Adapted DNA was PCR amplified, sonicated and prepared for Illumina sequencing. TaDa sequencing data were aligned to *Drosophila* genome annotation release 6.

Image Acquisition and Processing

Fluorescent images were acquired using a Leica SP8 confocal microscope. Images were analysed using Fiji software (Schindelin et al., 2012). Images were processed for brightness and contrast using Adobe Photoshop. Msh binding data were visualised using Integrative Genomics Viewer (IGV) software (Robinson et al., 2011). Figures were compiled in Adobe Illustrator.

QUANTIFICATION AND STATISTICAL ANALYSIS

NSCs in the tVNC of the *Drosophila* central nervous system were quantified throughout this study. Quantifications in the form 'NSCs per hemi-segment' are average values calculated by dividing the total number of NSCs in the tVNC by six (the number of hemi-segments in the tVNC). R was used for statistical analysis. Data were tested for assumptions of normality (Shapiro-Wilk test) and equality of variance (Levene's test). Statistical tests can be found in the relevant figure legends. Statistical significance was defined as $p<0.05$. No data were excluded.

Developmental Cell, Volume *49*

Supplemental Information

Dorsal-Ventral Differences in Neural Stem Cell Quiescence Are Induced by p57^{KIP2}/Dacapo

Leo Otsuki and Andrea H. Brand

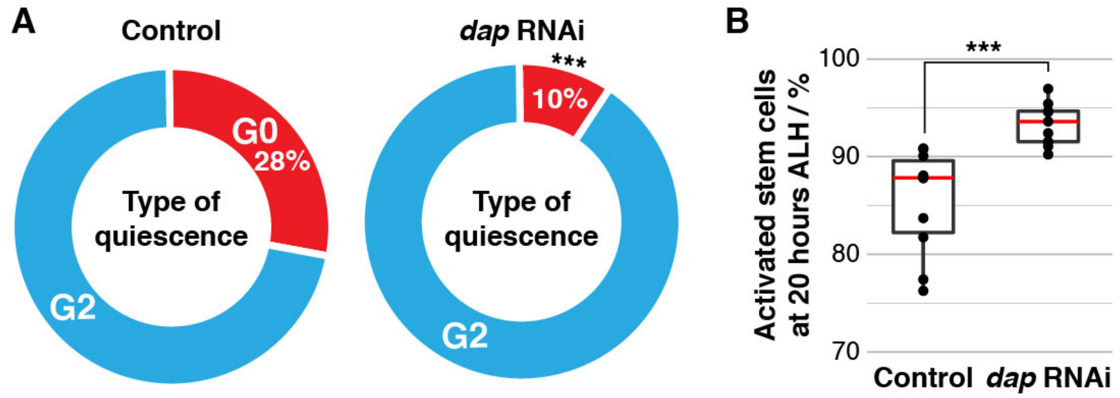

<u>Figure S1, Related to Figure 1. *dap* knockdown in NSCs leads to a switch from G_0 to G_2 quiescence and acceleration in stem cell activation timing</u>

(**A**) Proportions of G_0 and G_2 quiescent NSCs in control (*wor*-GAL4>*mCherry* RNAi) *versus* *dap* knockdown (*wor*-GAL4>*dap* RNAi) brains at 0ALH. Control: 28±1.0% G_0. *dap* knockdown: 10±0.5% G_0. *n*=10 tVNCs (control) or 7 tVNCs (knockdown), ~140 NSCs each. ***: $p=5.04\text{x}10^{-10}$, Welch's test.

(**B**) Activated NSCs in control (*wor*-GAL4>*mCherry* RNAi) *versus dap* knockdown (*wor*-GAL4>*dap* RNAi) tVNCs at 20 hours ALH. Wor expression was used to label activated stem cells (Otsuki and Brand, 2018). *n*=10 tVNCs (control) or 9 tVNCs (RNAi), ~130 NSCs each. ***: $p=7.65\text{x}10^{-4}$, Student's *t*-test.

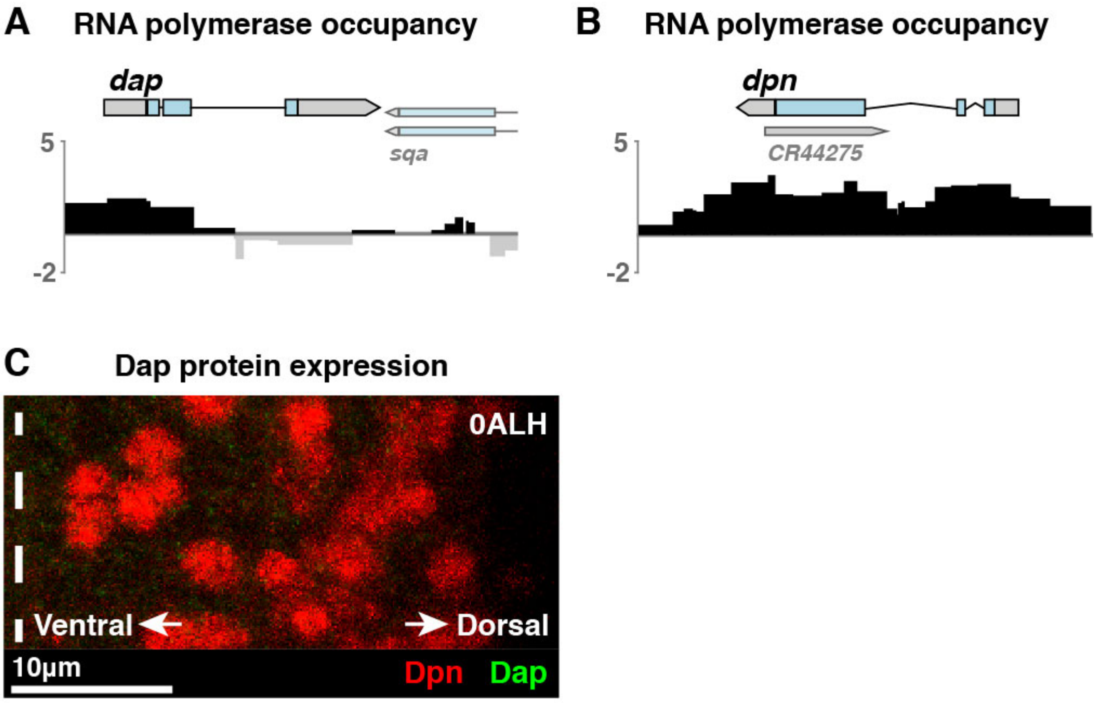

<u>Figure S2, Related to Figure 2. Quiescent NSCs do not transcribe or translate *dap*</u>

(**A**-**B**) RNA polymerase II occupancy at the *dap* and *dpn* loci in quiescent NSCs, as determined by Targeted DamID. RNA polymerase II is poised at the 5' end of the *dap* locus, but *dap* is not transcribed (A), in contrast to *dpn* which is actively transcribed in quiescent NSCs (B). Axis depicts log_2 ratio change between test and reference samples. Data from Otsuki and Brand, 2018.

(**C**) Quiescent NSCs do not express Dap protein at 0ALH. Single hemi-segment of a tVNC co-stained to visualise NSCs (red) and Dap (green) at 0ALH. Dotted line indicates the ventral midline. Anterior is up and dorsal is to the right. Maximum intensity projection.

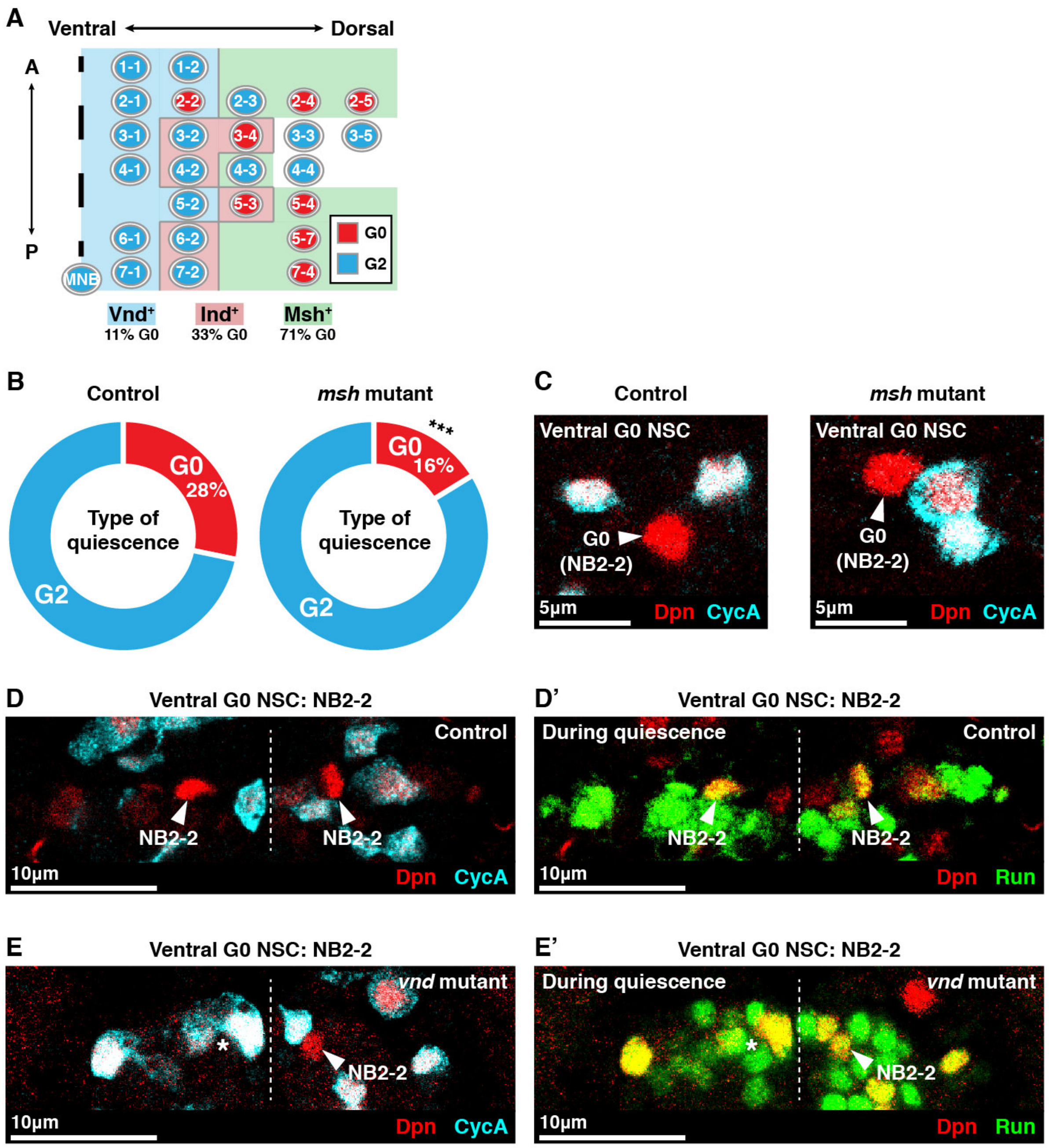

Figure S3, Related to Figure 3. Dorsal-ventral patterning of NSCs in the tVNC

(**A**) Map overlaying the distribution of G_0 (red) and G_2 (cyan) quiescent NSCs with the Msh$^+$ (pale green), Ind$^+$ (pale red) and Vnd$^+$ (pale blue) regions of the tVNC. Map assembled using data from (Chu et al., 1998; D'Alessio and Frasch, 1996; Isshiki et al., 1997; McDonald et al., 1998; Otsuki and Brand, 2018; Weiss et al., 1998). Dotted line indicates ventral midline. A: anterior. P: posterior.

(**B**) Proportions of G_0 and G_2 quiescent NSCs in control (*msh*$^{\Delta 68}$ heterozygous) *versus msh*$^{\Delta 68}$ mutant brains at stage 17 embryogenesis. *n*=10 tVNCs, ~140 NSCs each. ***: p=3.70x10^{-6}, Welch's test. Error bars indicate S.E.M.

(**C**) The ventral G_0 NSC NB2-2 (arrowed) still arrests in G_0 ($CycA^-$; red) in *msh* mutants. Single section confocal image.
(**D-D'**) In control embryos, the ventral G_0 NSC NB2-2 (arrowed) can be identified by its proximity to the midline (dotted line) and expression of the transcription factor Runt (Run, green). NB2-2 does not express CycA (cyan), identifying it as a G_0 NSC. Stage 17 embryo, single section confocal image.
(**E-E'**) NB2-2 is not always formed in *vnd*^{6} mutant embryos (Chu et al., 1998). However, in 33% of hemi-segments, a G_0 ($CycA^-$), Run^+ NSC is present close to the midline (dotted line), which we interpret to be the ventral G_0 NSC NB2-2. *n*=10 embryos, 6 hemi-segments each. Asterisk indicates expected position of NB2-2 in a hemi-segment lacking NB2-2. Stage 17 embryo, single section confocal image.
Anterior is up in all images.

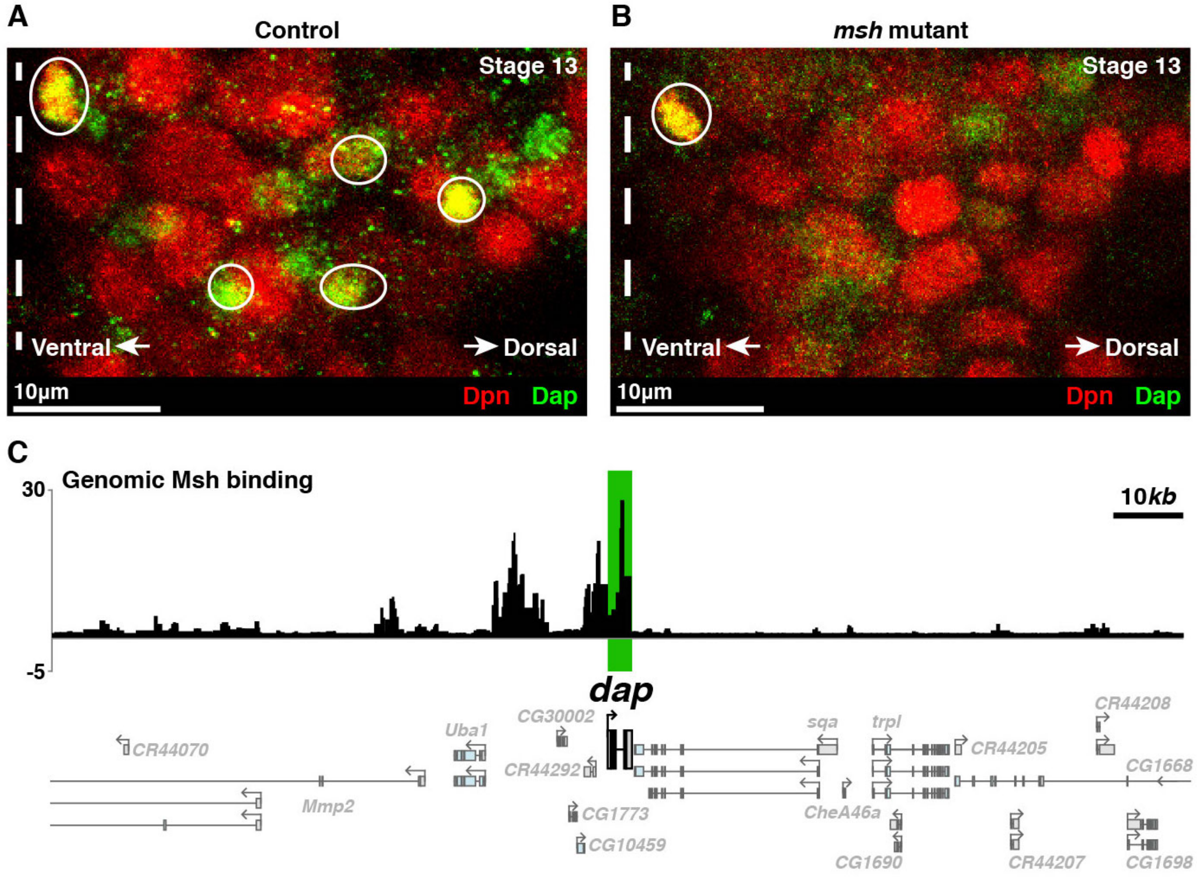

Figure S4, Related to Figure 4. Msh regulates Dap expression in NSCs

(**A**-**B**) Single hemi-segments from control (A) and *msh* mutant (B) tVNCs, co-stained to visualise NSCs (red) and Dap (green). Dap^{+} NSCs (yellow) are circled. Anterior is up; dorsal is right. Dorsal NSCs lose Dap expression in *msh* mutants. Dotted line indicates midline. Maximum intensity projections.

(**C**) Msh binding intensity across ~175kb of chromosome 2R, encompassing the *dap* locus (green box). Msh binds to the *dap* locus. Msh binding was assessed specifically in embryonic NSCs *in vivo* using TaDa (Southall et al., 2013). Unlogged data assembled from three biological replicates.
